# Efficacy and Effectiveness of Mobile Health Technologies for Facilitating Physical Activity in Adolescents: Scoping Review

**DOI:** 10.2196/11847

**Published:** 2019-02-12

**Authors:** Alexandra M Lee, Sarah Chavez, Jiang Bian, Lindsay A Thompson, Matthew J Gurka, Victoria G Williamson, François Modave

**Affiliations:** 1 Department of Health Outcomes and Biomedical Informatics College of Medicine University of Florida Gainesville, FL United States; 2 Department of Pediatrics, Division of General Pediatrics College of Medicine University of Florida Gainesville, FL United States; 3 Department of Psychology College of Liberal Arts and Sciences University of Florida Gainesville, FL United States; 4 Center for Health Outcomes and Informatics Research Health Sciences Division Loyola University Chicago Maywood, IL United States

**Keywords:** review, mobile health, adolescent, exercise

## Abstract

**Background:**

Increasing physical activity (PA) levels in adolescents aged 12 to 18 years is associated with prevention of unhealthy weight gain and improvement in cardiovascular fitness. The widespread availability of mobile health (mHealth) and wearable devices offers self-monitoring and motivational features for increasing PA levels and improving adherence to exercise programs.

**Objective:**

The aim of this scoping review was to identify the efficacy or effectiveness of mHealth intervention strategies for facilitating PA among adolescents aged 12 to 18 years.

**Methods:**

We conducted a systematic search for peer-reviewed studies published between 2008 and 2018 in the following electronic databases: PubMed, Google Scholar, PsychINFO, or SportDiscus. The search terms used included mHealth or “mobile health” or apps, “physical activity” or exercise, children or adolescents or teens or “young adults” or kids, and efficacy or effectiveness. Articles published outside of the date range (July 2008 to October 2018) and non-English articles were removed before abstract review. Three reviewers assessed all abstracts against the inclusion and exclusion criteria. Any uncertainties or differences in opinion were discussed as a group. The inclusion criteria were that the studies should (1) have an mHealth component, (2) target participants aged between 12 and 18 years, (3) have results on efficacy or effectiveness, and (4) assess PA-related outcomes. Reviews, abstracts only, protocols without results, and short message service text messaging–only interventions were excluded. We also extracted potentially relevant papers from reviews. At least 2 reviewers examined all full articles for fit with the criteria and extracted data for analysis. Data extracted from selected studies included study population, study type, components of PA intervention, and PA outcome results.

**Results:**

Overall, 126 articles were initially identified. Reviewers pulled 18 additional articles from excluded review papers. Only 18 articles were passed onto full review, and 16 were kept for analysis. The included studies differed in the sizes of the study populations (11-607 participants), locations of the study sites (7 countries), study setting, and study design. Overall, 5 mHealth intervention categories were identified: website, website+wearable, app, wearable+app, and website+wearable+app. The most common measures reported were subjective weekly PA (4/13) and objective daily moderate-to-vigorous PA (5/13) of the 19 different PA outcomes assessed. Furthermore, 5 of 13 studies with a control or comparison group showed a significant improvement in PA outcomes between the intervention group and the control or comparison group. Of those 5 studies, 3 permitted isolation of mHealth intervention components in the analysis.

**Conclusions:**

PA outcomes for adolescents improved over time through mHealth intervention use; however, the lack of consistency in chosen PA outcome measures, paucity of significant outcomes via between-group analyses, and the various study designs that prevent separating the effects of intervention components calls into question their true effect.

## Introduction

### Background

Mobile health (mHealth) is the use of mobile or wireless devices to support medical and public health practice [1]. mHealth leverages the availability of technological innovations such as biological sensors, short message service (SMS), Global Positioning System, and accelerometry that are small enough to be embedded into wearable devices and smartphones. These novel technologies provide an easy way to collect health-related data and allow consumers to monitor their own health data. mHealth offers modalities that are easily accessible and low-cost to implement, permitting potential reach across socioeconomic gradients and into hard-to-reach populations [2]. The mHealth market was projected to reach US $23 billion in 2017 and it is estimated to grow more than 35% in the next 3 years [3]. A large proportion of growth in mHealth is in the native app and wearable activity device market. Native apps are apps developed specifically for a smartphone device that can be directly downloaded onto the device platform from the app marketplace (eg, Apple App Store, Google Play store). Wearable activity devices collect information about the movement activity of the individual wearing the device, and some are compatible with native or Web apps (eg, FitBit). In 2017, there were over 325,000 health-related apps available in major app stores (eg, Apple’s App Store and Google’s Play Store) equating to 3.7 billion app downloads [4]. Smartphone use is widespread in US adults with 77% owning a smartphone; however, in adolescents, it is even more prevalent, with 94% owning or having access to a smartphone and 89% indicating that they access the internet *almost constantly* or *several times a day* [5].

### Behavior Change and Mobile Health

Emerging evidence shows that mHealth can aid in health behavior change resulting in better health outcomes, from smoking cessation and glucose monitoring to antiretroviral medication adherence and asthma control [6-8]. For successful prevention or management of many health conditions, health behavior changes are required, and a common recommendation has been to change physical activity (PA) behavior. Decreased PA levels are associated with several leading causes of death in the United States such as cardiovascular disease, cancer, and diabetes [9]. Previous reviews looking at the efficacy or effectiveness of mHealth for facilitating PA in adults have shown mixed evidence for the effectiveness of mHealth at increasing PA. One systematic review showed no impact on PA outcomes [10], and a meta-analysis presented a moderate effect on step counts and a nonsignificant effect on time in moderate-to-vigorous PA (MVPA) [11]. This lack of decisive findings could be attributed to low app quality and adherence to guidelines for exercise prescription [12] or a lack of variety in theories of behavior change employed by the app [13].

For the sake of clarity, in this paper, we use the term efficacy to discuss the performance of an intervention in a controlled environment, whereas effectiveness is understood as the performance of an intervention in a pragmatic setting. Nonetheless, these terms should not be viewed as binary attributes but rather as the range of performance as a continuous variable. Despite this mixed evidence as to the efficacy and effectiveness, understood in the context of continuity [14], of mHealth for facilitating PA in adults, there is a lack of evidence for an adolescent population despite its higher level of smartphone adoption and use. Adolescence is a sensitive period of neurocognitive development with effects on decision-making and behavior, making it a prime time for intervention regarding health-related behaviors. Though opportunities for prevention of chronic diseases can begin as early as the prenatal period, new health-related behaviors can arise in adolescence, making it a critical time point for prevention [15]. Vigorous PA levels decline by as much as 17.8% in boys and 11.0% in girls from middle to high school [16]. Increased knowledge about exercise, self-motivation, peer modeling and support, parental support, and availability of supplies or equipment are all positively associated with PA in adolescents [17,18]. mHealth modalities could address each of these correlates to PA. Although the meta-analysis [11] mentioned previously did include 2 studies within the adolescent age range, both studies incorporated SMS as the only mHealth intervention component. A systematic review of SMS interventions in youth and adolescents has recently been published [19]; thus, this review will focus on native and Web app interventions.

### Objectives

The aim of this review was to identify the efficacy or effectiveness of mHealth for facilitating adolescent PA. Scoping reviews permit quick structured mapping of key concepts in a research area, identify gaps in the existing literature, and succinctly summarize emerging research findings [20]. This scoping review is timely as there is no review in the adolescent age group regarding the efficacy or effectiveness of mHealth for PA. In addition, the mHealth market is rapidly growing; therefore, a quicker review process is ideal for dissemination that would be timely to researchers and clinicians looking to improve PA outcomes and provide relevant clinical guidance to patients.

## Methods

### Search Methods

We followed the scoping review methodology proposed by Arksey and O’Malley [20]. A scoping review involves 5 stages: (1) identifying the research question, (2) identifying relevant studies, (3) selecting the studies, (4) charting the data, and (5) collating the results (writing the manuscript) [20]. Two of the key differences between scoping reviews and systematic reviews lie in the search strategy and the assessment of evidence quality. Scoping reviews have broad research questions and invoke an iterative search process to identify all relevant articles [20]. They also do not seek to assess the quality of evidence for included studies [20]. In stage 1, we identified the following research question: Are mHealth interventions effective for increasing PA among adolescent populations? In stage 2, we identified databases, search terms, and set time constraints. We selected 4 primary databases for searching: PubMed, Google Scholar, PsychINFO, and SportDiscus. Although PubMed serves as the main foundation for publications within medical and public health journals, we added Google Scholar, PsychINFO, and SportDiscus to pull articles that could be in journals more relevant to software engineering, behavior change, and exercise science, respectively. The following terms were identified a priori by AML and FM and then entered into all 4 databases: mHealth or mobile health or apps, “physical activity” or exercise, children or adolescents or teens or “young adults” or kids, and efficacy or effectiveness. Due to differences in search engine functionality, the method by which terms were entered differed per database. See Multimedia Appendix 1 for detailed search methodology. Abstracts outside of the date range (July 2008-October 2018) and abstracts without articles available in English were removed before abstract review. The July 2008 start date marks the launch of the Apple App Store and the presence of smartphone apps in the consumer market. In stage 3, 3 reviewers (AML, SC, and FM) assessed all abstracts against the study criteria. Any uncertainties or differences in opinion were discussed as a group, and if unresolved in discussion, the full article was then reviewed. Inclusion criteria were (1) study participants aged 12 to 18 years, (2) address efficacy or effectiveness of mHealth, (3) mHealth component, and (4) PA-related study outcomes. PA study outcomes were considered to be measures of PA volume and health indicators representative of changes in PA, for example, cardiorespiratory fitness or strength. Exclusion criteria encompassed abstracts without a full article, protocols without results, interventions that only included SMS as the mHealth component, dissertations, or review papers. Reviewers searched all excluded review papers’ referenced articles for applicability to scope and added them to the abstract review if deemed plausible to fit. Once the abstract review was complete, the 3 reviewers (AML, SC, and FM) read each of the full articles to examine each article for fit with the criteria. As scoping reviews permit an iterative search process, any articles that were found outside of designated searches were sent to all 3 reviewers as a full article to be evaluated for inclusion into the review. Finally, in stage 4, AML, SC, and VW extracted data for analysis from articles that passed full review. Mendeley citation manager and Microsoft Excel were used to organize the references pulled from searches and to complete our assessment against the inclusion and exclusion criteria, respectively.

### Analysis

We charted the included studies according to key characteristics identified by the authors to delineate the efficacy or effectiveness of mHealth for facilitating PA. The key characteristics identified pre-extraction included year, location, number of participants, age, sex, race and/or ethnicity; study design, setting, duration; mHealth intervention components, additional intervention components, PA outcome measures, and PA outcome results. Setting indicates where recruitment, implementation, and measures of the study occurred. Duration identifies how long the mHealth intervention was used, and subsequent PA outcomes were tracked as previous behavior change research regarding PA has highlighted the difficulties with maintenance [21,22]. Charting the study design provided information on the rigor of the science and whether adequate comparisons were implemented as well as the strength of the conclusions that could be made. Descriptive statistics were calculated regarding the presence or absence of PA characteristics among adolescents in the included studies.

## Results

**Figure 1:**
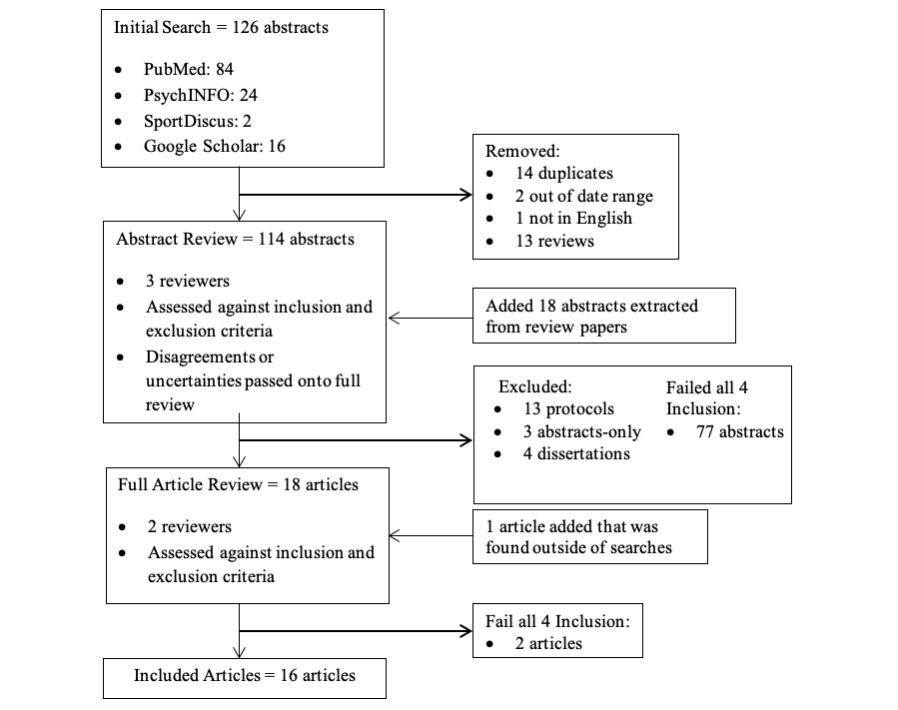
Flowchart of search methods and exclusion process.

Through our database searches, we initially identified a total of 126 abstracts, of which, 14 were duplicated. Overall, 2 articles were removed as the studies were performed outside the date range of interest, and 1 article that was not in English was excluded as well. In addition, we removed 13 review articles from which we extracted 18 papers of interest for this scoping review, leading to a total of 96 distinct articles. Three reviewers assessed the 88 abstracts for inclusion and exclusion. Abstracts that led to disagreement and/or uncertainty of relevance were included in full review. Overall, 13 articles were protocols, 3 only had an abstract, 4 were dissertations, and 77 failed our inclusion criteria. A total of 17 articles were then considered for full review. Finally, 2 articles were excluded after full review as they did not meet the inclusion criteria. An additional article identified at the end-stage review was sent through full-article review and added to the analysis, totaling 16 studies (see Figure 1). Descriptions of each of the included studies are found in Table 1.

### Participants

The 16 included studies differed greatly in size (11-607 participants) and location (7 countries). Of them, 8 studies [23-30] took place in the United States and provide varying breakdowns of racial and/or ethnic groups. For each of the main races and/or ethnicities in the United States (white, Hispanic, black, or Asian), there was at least 1 study per group that sampled them in the majority.

### Design and Setting

The included studies comprised 9 randomized controlled trials [23-26,30-34], 1 comparative effectiveness trial [28], 3 quasi-experimental studies [35-37], and 3 not randomized or controlled studies [27,29,38]. The most common recruitment setting was at the school (8/16) [28,29,32-36,38], followed by primary care (4/16) [23,26,27,37] and the community (4/16) [24,25,29,31]. Most implementation occurs in a home environment; however, the settings for measurements are split among home, university, school, and primary care.

### Study Components

Study components and PA outcomes are described in Table 2. Primary mHealth intervention components for all included studies fell into the following categories: wearables, apps, and websites, and about half of the studies utilized 1 component and the other half used 2 or more mHealth components. The 5 mHealth intervention category combinations utilized were website [24,28,35,37], website+wearable [25,26,29,33,36], app [31,32,38], wearable+app [23,27], and website+wearable+app [30,34]. The most common singular mHealth component is a website. No studies used a wearable alone; however, it was the most common component to be paired with another mHealth component. Overall, 10 out of 16 studies [23-26,28, 30,31,33,37,38] isolated mHealth components as the sole intervention method. *Isolate* refers to whether the intervention either solely included mHealth intervention components (versus *additional intervention components*, as described in Table 2) or the design of the study permitted an analysis that could look at the individual effect of the mHealth component on the PA-related outcome variable(s). Additional intervention components ranged from goal-setting guides to educational seminars or group counseling and skills training. In the 12 controlled studies [23-26,30-37], 8 control groups were simply advised to continue usual care or normal PA behaviors [26,30-32,34-37].

**Table 1:** Description of included studies.

Author (Year), Country	N	Age (years), mean (SD) or range	Study design, duration, and arms	Setting
Chen et al^a^ (2017) [23], USA	40	15 (1.56)	RCT^b^; 3 months; Intervention and control	Primary care (recruitment); Home (implementation); Web-based (measures)
Cullen et al (2013) [24], USA	291	12-16	RCT; 8 weeks; Intervention and control	Community (recruitment); Home (implementation); Measurement unclear
Direito et al (2015) [31], New Zealand	51	15.7 (1.2)	RCT; 8 weeks; interventions and 1 control	Community (recruitment); Home (implementation); University (measures)
Gaudet et al (2017) [36], Canada	23	13 (0.3)	Quasi-experimental crossover; 7 weeks; Immediate intervention and delayed intervention (each served as control)	School (recruitment); Home (implementation); Home (measures)
Guthrie et al^a^ (2015) [25], USA	182	12.7 (0.9)	RCT; 6 weeks; Active control, passive control, and intervention	Community (recruitment); Home (implementation); Measures unclear
Jimoh et al (2018) [38], United Kingdom	34	16-19	Not randomized or controlled; 8 weeks (4 with paper diary and 4 with app)	School (recruitment, implementation); Home (implementation and measures)
Kennedy et al^a^ (2017) [32], Australia	607	14.1 (0.5)	Cluster RCT; 10 weeks; Matched pairs randomization at school level to intervention or control	School (recruitment, implementation and measures); Home (implementation)
Larsen et al (2018) [29], USA	21	14.7 (2.1)	Not randomized or controlled; 12 weeks	School (recruitment); Community (recruitment); Home (implementation)
Lau et al (2012) [35], Hong Kong	78	12-15	Quasi-experimental; 8 weeks; Intervention and control	School (recruitment, measures); Home (implementation)
Mendoza et al (2017) [30], USA	59	16.6 (1.5)	RCT; 10 weeks; Intervention and control	Survivor database and clinic (recruitment); Home (implementation and some measures)
Patrick et al (2013) [26], USA	101	14.3 (1.5)	RCT; 12 months; 3 interventions and 1 control	Primary care (recruitment, some implementation, and measures); Home (implementation)
Schoenfelder et al (2017) [27], USA	11	15.5 (1.4)	Not randomized or controlled; 4 weeks	Primary care (recruitment); Home (implementation and measures)
Slootmaker et al^a^ (2010) [33], the Netherlands	87	13-17	RCT; 3 months; Intervention and control	School (recruitment and measures); Home (implementation)
Smith et al^a^ (2014) [34], Australia	361	12.7 (0.5)	Cluster RCT; 7 months; School-level matched pairs randomization to intervention or control	School (recruitment, measures, and implementation)
Sousa et al (2015) [37], Portugal	94	12-18	Quasi-experimental; Pre-post design with control; 24 weeks	Clinic (recruitment, implementation, and measures); Home (implementation)
Whittemore et al (2013) [28], USA	384	15.31 (0.69)	Cluster RCT; Class level; 6 months; Comparison of 2 interventions	School (recruitment, implementation, and measures)

^a^Significant between-group results.

^b^RCT: randomized controlled trial.

**Table 2:** Study components and physical activity outcomes.

Study	Control group	mHealth intervention component	Additional intervention component	Physical activity outcomes	Results
Chen et al^a^ (2017) [23]	Given Omron HJ-105 pedometer+blank food diary+online program with 8 modules on general adolescent health	FitBit flex+iStart Smart mobile app	None	Number of days per week with 60 min MVPA^b^ (S)	Significant time X group interaction for reported days of PA^c^. Medium effect size
Cullen et al (2013) [24]	Online program with educational materials about nutrition and PA, printable goal sheets	Online program with educational materials about nutrition and PA, role model stories, online self-monitoring, goal review, and problem-solving components	None	60 min of PA on 5 days per week; (S^d^); 60 min of PA 7 days per week; (S)	Post intervention, significantly more adolescents reported being physically active for >60min/day on all 7 days (*P*<.001); 84% of adolescents reported online program was helpful for increasing PA
Direito et al (2015) [31]	Asked to continue normal PA	Immersive app: Zombies Run! 5K training; Nonimmersive app: Get Running-Couch to 5K	None	Cardiorespiratory fitness (O); Time to run 1 mile (O); Weekly PA (S); Daily MVPA (O)	No difference in VO_2_ peak between interventions and control. No intervention effect for self-reported PA or objective MVPA. Group assignment did not have significant effect on mean daily MVPA. For those that used app 3 times/week, statistically significant decline in time to run for nonimmersive app compared with control
Gaudet et al (2017) [36]	Nothing given	FitBit Charge HR and FitBit Web app	Taught SMART and told to set goals	Daily MVPA (O); Step Counts (O)	The immediate intervention group A increased MVPA by 10.9 min (*P*=.03) during their intervention but Group B did not significantly increase during their intervention. There were no significant differences in MVPA between groups at any time point. Students at baseline that were in the adoption phase experienced a significant increase in MVPA by more than 15 min/day
Guthrie et al^a^ (2015) [25]	Active: Received Zamzee activity monitors and Dance Revolution video game; Passive: Received Zamzee activity monitors	Zamzee activity monitors+website with monitor feedback and incentive motivation	None	Daily MVPA (O)	MVPA duration differed significantly across groups (*P*<.01). Intervention group showed an average 15.26 min/day MVPA, which is 49% greater than passive control (*P*<.01) and 67% greater than active control (*P*<.01)
Jimoh et al (2018) [38]	No control	Smartphone app diary for food and exercise recording; SMS^e^ with personalized feedback	Paper diaries; In-person meetings with research team to review paper diaries every 2 weeks	Weekly PA (S)	No significant differences between app and paper diary in reported volume of aerobic and strength training during respective intervention period
Kennedy et al^a^ (2017) [32]	Standard school procedures	Web-based smartphone app	Interactive student seminars, structured PA program, lunch-time fitness sessions	Upper body muscular endurance (O^f^), lower body muscular strength (O), Daily MVPA (O), Cardiorespiratory Fitness (O), RT^g^ skill competency(O)	Significant intervention effects for upper body muscular endurance at 6 and 12 months; Significant group-by-time interactions for estimated VO_2_ max at 6 months; No significant intervention effects for weekday MVPA. Significant group-by-time effects for RT^g^ skill competency at 6 and 12 months
Larsen et al (2018) [29]	No control	Website with activity manuals matched to current level of motivational readiness, tailored reports based on regular assessment, tip sheets, local activity resources; Pedometer	One-on-one goal-setting session; Tip sheets for moms of participants	Weekly MVPA (S)	Weekly MVPA increased from 24.7 min at baseline to 79.4 min at follow-up (*P*<.01). Each participant increased an average of 58.8 min/week (*P*<.01).
Lau et al (2012) [35]	Nothing given	Internet PA program (behavioral skill training, self-monitoring, tailored feedback, PA planner, chat room; SMS; (virtual friend; message types: behavioral skills, reinforcement of PA benefits, solutions for PA barriers, motivational, informational)	N/A^h^	Weekly PA (S)	Significant increase in intervention group (*P*=.05) but not in control group (*P*=.34); however, the intergroup differences were not significant
Mendoza et al (2017) [30]	Usual Care	FitBit Flex and Fitbit app; Facebook group-team and individual motivation badges from staff, and participant discussion of shared experiences; Phone/SMS 1/week to set goal; SMS every other day for reminder and encouragement	N/A	Daily MVPA (O)	Within-group changes in PA from baseline to follow-up not reported. No significant difference in daily MVPA between the intervention and the control group.
Patrick et al (2013) [26]	Usual care	All included: Pedometer; Web only group: program website with Web tutorials+weekly check-in emails; Web+group sessions: program website with Web tutorials; Web+SMS: program website, 3 text messages/week (content, reminders, or questions to counselor)	All 4 conditions included: monthly mailed tip sheets; Web only: also included weekly email check-ins; Web+group also included monthly 90 min group behavioral skills sessions and bimonthly phone calls from a health counselor	Weekly MVPA (S)	No significant differences observed for any PA outcomes
Schoenfelder et al (2017) [27]	No control	FitBit flex+FitBit mobile app; Facebook group; Daily SMS	None	Step Counts (O)	Daily step counts averaged 8014. From baseline to 4 weeks, step count significantly increased by 3218 steps (*P*<.01)
Slootmaker et al^a^ (2010) [33]	Received brochure with general PA recommendations	PAM accelerometer+PAM coach website	None	Weekly PA (S)	Girls in intervention group significantly increased moderate PA after 3 months (*P*=.04) compared with control, but effect disappeared at 8 months.
Smith et al^a^ (2014) [34]	Standard school procedures	Pedometers; Smartphone app and website	Teacher professional development; Parent newsletters; Researcher-led seminars; Enhanced school sport sessions; Lunchtime PA-mentoring sessions	Weekly PA (O); Weekly MVPA (O); RT Skill competency (O); Upper body maximal strength and endurance (O)	No significant differences in overall PA counts or MVPA; Significant intervention effect on upper body muscular endurance (*P*=.04) and RT skill competency (*P*<.01)
Sousa et al (2015) [37]	Standard treatment program of clinical evaluation, medical, psychological, nutritional, and PA counseling	Next Step: e-therapeutic platform (education, self-monitoring, social support, interactive training, and motivational tools)	Standard treatment program of clinical evaluation, medical, psychological, nutritional, and PA counseling	Weekly PA (S)	Significant improvement in PA in intervention group (*P*<.03)
Whittemore et al (2013) [28]	Comparative effectiveness	HEALTH[e]TEEN website (lessons, goal setting, self-monitoring, health coaching, and social networking)	CST-coping skills training, combined with website for one of the intervention groups	Days per week 60 min MVPA. (S); Days per week of muscle strengthening (S)	In both groups, adolescents significantly increased MVPA over 6 months. No time effects for muscle strengthening.

^a^Significant between-groups results.

^b^MVPA: moderate-to-vigorous physical activity.

^c^PA: physical activity.

^d^S: subjective measure.

^e^SMS: short message system.

^f^O: objective measure.

^g^RT: resistance training.

^h^N/A: not applicable.

### Physical Activity Outcomes

The PA outcomes reported can be divided into 5 categories: days per week meeting activity guidelines, cardiorespiratory fitness, MVPA, general activity, muscular strength, and competency. Within each of these categories, individual measures can be isolated by the amount of time they capture and whether they are subjective or objective measures. Parsed out, there are 18 total PA outcomes measured among the 16 studies included (see Table 1, column titled *Physical Activity Outcomes*). A total of 8 measures appear in more than 1 study, the most common being objective daily MVPA (5/13) [25,30-32,36] followed by subjective weekly PA (5/16) [31,33,35,37,38]. Of the 18 PA outcome measures, 44% were subjective, self-reported measures. Significant improvement in a PA outcome over time in the group with the mHealth intervention was observed for all but 2 of the eligible included studies (ineligible: Direito et al and Mendoza et al, which did not present results for this type of analysis); however, this improvement was not always unique to the intervention group. Of the 13 studies that contained a control or comparison group, only 5 [22,24,31,32,33] showed an improvement in a PA outcome that was significantly different from the results of the control or comparison group. Within the 5 studies that showed significant results for between-groups analyses, 3 studies [22,24,32] isolated the mHealth component in the intervention. In addition, 2 of the 3 studies utilized a website and wearable intervention [24,32] and 1 utilized an app and a wearable [23]. The PA outcomes that improved in the 3 studies were objective daily MVPA [25], subjective weekly PA [33], and number of days per week of 60 min of PA [23], respectively.

**Table 3:** Mobile health intervention components and significant improvement in physical activity outcomes.

Study and modality		Duration ≥12 weeks	Increasing PA^a^ outcome over time in experimental group or all groups	Increasing PA outcome between groups or group x time	Isolated the mHealth component
**Website**					
	Lau et al (2012) [35]		✓ Weekly MVPA^b^ (S^c^)		
	Cullen et al (2013) [24]		✓60 min PA 7 days/week (S)		✓
	Whittemore et al (2013) [28]	✓	✓ # days/week meeting MVPA rec (S)		✓
	Sousa et al (2015) [37]	✓	✓ Weekly PA (S)		✓
**App**				
	Direito et al (2015) [31]		Not reported		✓
	Kennedy et al (2017) [32]		✓ Upper body muscular endurance (O^d^), cardiorespiratory fitness (O), RT^e^ skill competency (O)	✓ Upper body muscular endurance (O), cardiorespiratory fitness (O), RT skill competency (O)	
	Jimoh et al (2018) [38]			Not applicable	✓
**Wearable+Website**					
	Slootmaker et al (2010) [33]	✓	✓ Weekly PA (S)	✓ Weekly PA (S)	✓
	Patrick et al (2013) [26]	✓			✓
	Guthrie et al (2015) [25]		✓ Daily MVPA (O)	✓ Daily MVPA(O)	✓
	Gaudet et al (2017) [36]		✓ Daily MVPA (O)		
	Larsen et al (2018) [29]	✓	✓ Weekly MVPA (S)	Not applicable	
**Wearable+App**				
	Schoenfelder et al (2017) [27]		✓ Step count	✓ Not applicable	
	Chen et al (2017) [23]	✓	✓ # days/week 60 min PA (S)	✓ # days/week 60 min PA (S)	✓
**Wearable+Website+App**					
	Smith et al (2014) [34]		✓ Upper body muscular endurance (O) and RT skill competency (O)	✓ Upper body muscular endurance (O) and RT skill competency (O)	
	Mendoza et al (2017) [30]		Not reported		✓

^a^PA: physical activity.

^b^MVPA: moderate-to-vigorous physical activity.

^c^S: subjective measure.

^d^O: objective measure.

^e^RT: resistance training.

A total of 5 different groupings of mHealth components in the intervention groups (app, wearable+app, website, website+wearable, and website+wearable+app) among 16 studies prevent this review from being able to identify a specific mHealth component as most effective for promoting PA in adolescents. Furthermore, research designs from included interventions did not always isolate the mHealth component for analysis on its sole effect on PA outcomes. The 3 studies that showed significant intervention effects on a PA outcome and isolated the mHealth component in the intervention included 1 study with a wearable+app and 2 studies with a website+wearable. Finally, we considered interventions with a duration longer than 12 weeks as this is frequently observed as the break-up point with respect to adherence. See Table 3 for a visual summary of mHealth components and corresponding improvement in PA outcomes.

Interventions included multiple therapeutic modalities to improve health outcomes, which makes it difficult to disentangle the separate effects for each modality. The extreme diversity in PA outcomes reported across studies also makes comparison among mHealth components challenging. Table 3 expands upon Table 2 and presents a visual representation of PA outcomes that significantly improved compared with the mHealth modality implemented. The main reasons for diversity of PA outcomes reported were each measure’s time unit and whether it was a subjective or objective measure. Roughly half of the measures were subjective, self-reported measures, and a systematic review by Adamo et al showed that self-reported PA data in a pediatric population are often overestimated compared with objective measures of PA, thus caution should be exhibited when interpreting these results [39].

## Discussion

### Principal Findings

This scoping review synthesizes intervention findings from 16 studies that measured the efficacy or effectiveness of mHealth to improve PA outcomes in adolescents. Our review identified 10 randomized control trials, 3 of which are cluster trials, 3 quasi-experimental interventions with control, and 3 studies without a control group. An initial observation indicates that interventions with an mHealth component could lead to general improvement in PA outcomes over time, as was observed in 12 of the 16 studies. However, when specifically considering studies with a control group, only 5 of 13 showed a significant intervention effect, and an additional 5 of 13 showed improvement among all groups. Of the studies with a control group and objective assessments of PA outcomes, 3 showed statistically significant improvements in the intervention groups compared with the control groups.

It is particularly interesting to see that a majority of the studies included in this scoping review are randomized controlled trials or cluster randomized controlled trials, which are evidently far stronger interventions to demonstrate effect significance. Therefore, despite the small number of studies with a significant intervention effect and objective measurements of PA, we do not think that this scoping review indicates that mHealth interventions are inappropriate to be used to improve PA among adolescents. However, we believe that this forces us to rethink how to use mHealth and how we build mobile apps. Given the growing body of literature showing the impact of mHealth interventions on behavior change [2,6,40,41], we surmise that this is more a reflection of the quality of the mobile apps [12,42-44] and their appropriateness to the specific population we are considering. There is reasonable evidence that apps developed in collaboration with their potential users are more likely to be used and to be effective [45,46]. Therefore, mHealth researchers interested in PA in adolescents should consider building their interventions with their target population from the very beginning. In this review, the study by Chen et al [23] was one of the few studies that found an intervention effect and the only one to create an adolescent stakeholder group for intervention development. This is particularly important given the rapid growth of app development. The company *Flurry* tracks app usage across platforms (iOS and Android). It has observed a 330% growth in health and fitness app usage over the past 3 years [47]. In the meantime, Statista reports that the global mHealth market grew from $21.1 billion in 2016 to $40 billion in 2018 and is expected to reach a staggering $332.7 billion by 2025 [48].

Interestingly, 3 of the 13 controlled studies looked specifically at improvements in muscular strength and conditioning, and 2 of them showed significant improvement. One could conjecture that strength training faring better than aerobic conditioning is simply a reflection of preferences of the specific population under study, particularly boys. Unexpectedly, there was a lack of interventions that leveraged *gamification*, for example, Pokémon GO–type approaches, or competition-type interventions using apps, such as Strava (Strava.com), that allow asynchronous competition in a social media environment. Pokémon GO has been shown to significantly increase the number of young adults who reach 10,000 steps per day [49], and some research shows that gamification could be appealing to adolescents [50].

Recruitment settings mirrored common sites for recruitment in intervention trials that have pediatric or adolescent populations, but the portability of mHealth interventions permitted most studies to use home-based implementation. Home-based implementation reduces participant transportation burden and cost. Measures were spread among 4 different sites, but this was because many of the included studies incorporated biological measures that cannot be measured on the Web or through an app, such as cardiorespiratory fitness and lower body muscular strength. Less than half of the studies were of a duration longer than 12 weeks, thus it is difficult to discern any long-term behavior changes as most studies did not extend far enough to evaluate PA outcome maintenance beyond the usual 12-week end point.

mHealth intervention components utilized within the studies included apps, wearables, and websites. The wearable is the only mHealth component that did not function as a solo intervention component but was always paired with an app or website. Innovations in technology have resulted in most new wearables pairing with an app or website that provides data feedback to the user, with some permitting a social network component, which could explain this observation.

### Strengths and Weaknesses

A limitation of scoping reviews is that because of their broad and less targeted approach compared with a traditional systematic review, quantitative results cannot be pooled to understand the effect size of mHealth technologies on PA outcomes in adolescents. However, the purpose of a scoping review methodology is to be broad reaching [20], which is appropriate in the context of mHealth and PA interventions in adolescents. A second limitation arises from the search methodology. There is a possibility that because of different databases requiring modifications of search methods, relevant studies may have been missed. This is primarily a concern with our Google Scholar search as the search engine would not adhere to our Boolean arguments, requiring large amounts of individual title sorting by 1 reviewer (AML). In addition, scoping reviews do not seek to assess the quality of evidence or provide a grading of recommendations assessment, development and evaluation for the included studies; therefore, we cannot comment on the strength or generalizability of the findings [20]. A strength of this review is that it provides a quick snapshot of research conducted in the last decade in the adolescent target population and includes several different kinds of mHealth tools that are employed in intervention work. Even though there have been reviews pertaining to the use of mHealth and PA among teens, to the authors’ knowledge, this paper is the first review to summarize newer tools, such as app and wearable interventions, on PA outcomes for adolescents. This is particularly important given the pace at which technology evolves especially with respect to accuracy of measurements and integration.

### Conclusions

In conclusion, the use of mHealth to improve PA in adolescents is an emerging field, complicated by frequent change in the rate at which new technologies develop. There is limited research about the efficacy and effectiveness of each mHealth modality, and future research designs need to provide avenues for analysis of the effect of the mHealth component alone on PA outcomes in adolescents in addition to the combined effect of the total intervention, if applicable. As there is a diversity of mHealth modalities, a reported component analysis of the specific modality utilized in a study, for example, an app, could provide information to compare it with other apps utilized in other studies. For example, apps can have social networking components, notifications, reward systems, gaming features, or education modules, all of which could affect PA outcomes differently; therefore, future work could be improved if these qualities were mapped out for comparison. In this sense, mHealth modalities could be evaluated as efficacious or effective on the basis of component analysis versus referring to their general category such as *website*, *app*, or *wearable*.

## Appendix

Multimedia Appendix 1 Detailed search methods per database.

## Abbreviations

mHealth: mobile health
MVPA: moderate-to-vigorous physical activity
PA: physical activity
SMS: short message service
