# Supplementary material for: Efficacy and Effectiveness of Mobile Health Technologies for Facilitating Physical Activity in Adolescents: Scoping Review
Source: JMIR Mhealth Uhealth. 2019 Feb 12;7(2):e11847. doi: 10.2196/11847 (PMC6390191; doi:10.2196/11847)
Supplement: Multimedia Appendix 1 [file mhealth_v7i2e11847_app1.docx]

## Multimedia Appendix 1

### Detailed Search Methods per Database

### PubMed

The following terms were entered as is into the search bar:

(mHealth OR “mobile health” OR apps) AND (“physical activity” OR exercise) AND (children OR adolescents OR teens OR “young adults” OR kids) AND (efficacy OR effectiveness)

### PsycINFO & Sport Discus


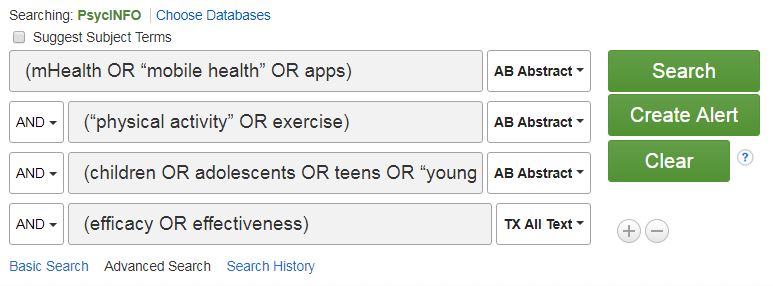


The image shows how search terms were entered to search in PsycINFO and SportDiscus. When all terms were set to “AB Abstract”, relevant articles based on title were cut from search results, and contrastingly, when all terms were set to “TX All Text”, search results were too numerous and outside of relevant subject area. The selected search strategy was identified through manipulation of search areas until the results, when broadened by one indicator, did not add any additional articles within the scope of the research question.

###
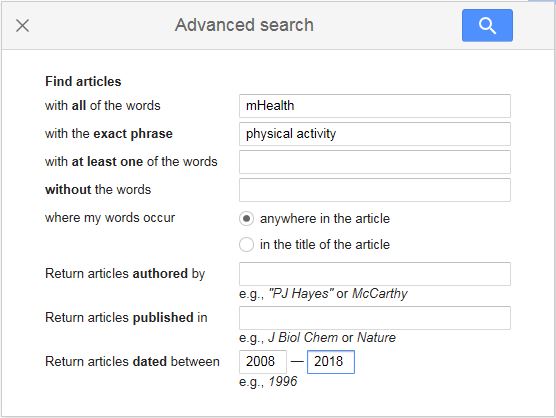
Google Scholar

The Advanced Search option was used to guide the search strategy for Google Scholar. The image to the right provides the backbone for all searches and the following combinations of terms was rotated through the first and second lines, respectively: mHealth, physical activity; mHealth, exercise; “mobile health”, physical activity; “mobile health”, exercise; app, physical activity; app, exercise. Abstracts were downloaded for any articles that appeared relevant by title up to the first 100 results.
